# An integrative mendelian randomisation and drug mechanism framework for target prioritisation and therapeutic repurposing in major depression

**DOI:** 10.1038/s41398-026-04137-9

**Published:** 2026-06-02

**Authors:** Abigail R. ter Kuile, Chris Finan, Sandesh Chopade, Marion van Vugt, Nikita Hukerikar, Serena Barral, Argyris Stringaris, Amand F. Schmidt, Karoline Kuchenbaecker, Jean-Baptiste Pingault

**Affiliations:** 1 Department of Clinical, Educational and Health Psychology, University College London, London, UK; 2 Institute of Cardiovascular Science, University College London, London, UK; 3 The National Institute for Health Research University College London Hospitals Biomedical Research Centre, University College London, London, UK; 4 Department of Cardiology, Division of Heart and Lungs, University Medical Centre Utrecht, Utrecht University, Utrecht, the Netherlands; 5 Department of Cardiology, Amsterdam Cardiovascular Sciences, Amsterdam University Medical Centre, University of Amsterdam, Amsterdam, the Netherlands; 6 Institute of Health Informatics, University College London, London, UK; 7 Developmental Neurosciences, Zayed Centre for Research into Rare Disease in Children, UCL GOS Institute of Child Health, University College London, London, UK; 8 Division of Psychiatry, University College London, London, UK; 9 Social Genetic and Developmental Psychiatry, King’s College London, London, UK

**Keywords:** Genomics, Depression, Clinical pharmacology

## Abstract

Major depression (MD) treatments have limited efficacy and target few mechanisms, highlighting the need for innovative drug discovery. Drugs targeting genetically supported proteins are 2.6 times more likely to succeed in drug development. Here, we use genetic methods to identify and prioritise MD drug targets, leveraging genome-wide association study (GWAS) summary statistics from >525,000 MD cases. We derived exposure data from 10 datasets measuring protein quantitative trait loci (pQTLs) and gene expression levels (eQTLs) in blood, cerebrospinal fluid, and brain tissues. We performed *cis*-Mendelian randomisation (MR) on 3469 druggable targets (genes encoding proteins targeted by existing compounds or experimentally predicted to be druggable). To strengthen causal inference, we implemented robust MR estimators, colocalisation, external replication, and assessed directional consistency across tissues. We integrated *cis*-MR effect directions with drug mechanisms and clinical annotations to infer potential therapeutic effects. Validation analyses showed that 82% of drugs approved for depression/anxiety had ≥1 significant MR target, compared to 51% for compounds in clinical trials. For repurposing, we prioritised 54 targets of compounds developed for other conditions with estimated beneficial effects on MD (e.g., an inhibitor for a risk-increasing target). Ten high-priority targets of brain-penetrating compounds included ACE and NISCH (cardiovascular drugs), NDUFA2, NDUFB6, and NDUFS1 (metformin), CDK4, NTRK3, and MET (oncology inhibitors), and GLS and NOS2 (enzyme inhibitors). We found genetic evidence for established and novel MD targets across the drug development pipeline. Novel targets point to mechanisms beyond monoaminergic systems, most with approved drugs for other conditions, offering immediate repurposing opportunities.

## Introduction

Major depression (MD) is a leading cause of years lived with disability globally [1]. Current pharmacological treatments are ineffective for many patients, with only one-third reaching remission after first-line antidepressants and 28–40% classified as having treatment-resistant depression [2, 3]. Currently approved drugs target a narrow range of therapeutic mechanisms, with most antidepressants acting on monoaminergic neurotransmitter systems [4]. The lack of novel pharmacological treatments for MD highlights the pressing need for innovative strategies to facilitate efficient drug discovery.

Human genetics offers a promising avenue to guide the development of pharmacological treatments. Drugs acting on targets with supporting human genetic evidence are 2.6 times more likely to succeed in drug discovery pipelines and progress into clinical practice than those without [5–7]. This is reflected in recent FDA approvals: 63% of drugs approved in the past decade had genetic support [8]. Given that genes encode proteins, which constitute over 90% of drug targets, genetic methods can identify disease-associated protein targets for repurposing existing drugs or novel development [9, 10]. To accelerate discovery and repurposing, an efficient strategy involves focusing on the ‘druggable genome’ rather than all protein-coding genes, reducing the multiple testing burden [9–11]. The druggable genome comprises genes encoding proteins targeted by drugs that are already approved, those at the clinical stage of drug development, or those potentially druggable based on experimental evidence [9].

Mendelian randomisation (MR) enables scanning of the druggable genome by using genetic variants as instruments to estimate the effect of altering each drug target’s activity on disease risk [12]. For each target protein, genetic variants selected as instruments lie near the protein-encoding gene (*cis*-region) [11]. These instruments are associated with individual variation in a molecular trait in a given tissue, including changes in protein values or mRNA expression [10]. *Cis*-MR leverages data from genome-wide association studies (GWAS) to obtain the effects of each selected variant on both the exposure (target protein activity) and the outcome (MD). To infer exposure-outcome causal effects, MR assumes that variant effects on the outcome operate through the exposure and are independent of confounders [12]. Under these assumptions, significant *cis-*MR estimates suggest modulating the corresponding proteins may influence disease processes, identifying candidate targets for that outcome [10, 12]. MR has key advantages for identifying drug targets. First, the random assortment of genetic variants at conception mimics treatment allocation in randomised controlled trials, strengthening causal inference of target-outcome effects [13]. Second, *cis-*MR provides effect directions to infer whether pharmacological activation or inhibition of a target mitigates disease risk [14]. Effect directions can be combined with pharmacological data on existing drugs to identify target-compound pairs with aligned mechanisms, aiding repurposing [14, 15].

The latest Psychiatric Genomics Consortium (PGC) Phase 3 MD GWAS provides unprecedented opportunities for well-powered drug target MR, identifying 635 genomic loci associated with MD [16]. Drug target enrichment analyses using sets of target-encoding genes and grouping them by drug classes showed that antidepressant and antipsychotic classes were enriched for MD-associated variants [16]. Eighteen individual compounds approved mainly for indications other than depression were enriched, including antipsychotics, treatment for cancer, anxiety (pregabalin), and narcolepsy (modafinil) [16]. However, as noted by the authors, enrichment analysis is not directional and cannot estimate whether compounds increase or decrease MD symptoms [16], highlighting the need for directional drug target MR.

While initial druggable genome *cis*-MR applications to MD have provided a foundation by inferring directional effects [17, 18], opportunities remain to advance this approach. We introduce an integrated approach leveraging methodological advances to robustly identify and prioritise targets. First, we enhance statistical power using the PGC Phase 3 MD GWAS [16]. Second, we expand the scope of the druggable genome beyond established targets to include novel candidates with experimental evidence of druggability. Third, we evaluate target effects across blood, cerebrospinal fluid (CSF), and multiple brain regions, beyond previous brain-restricted analyses [17, 18]. Finally, our framework strengthens the validation of genetic evidence by employing multiple robust MR estimators, colocalisation, external replication, and tissue-specific directional consistency.

To identify a tractable set of high-priority targets from our *cis*-MR findings for experimental and clinical validation, we integrate genetic evidence with clinical and pharmacological data. Such a framework has been applied to other non-psychiatric complex diseases, evaluating genetic robustness, clinical relevance, and pharmacological properties of target-compound pairs [14, 15, 19]. Aligning genetic effects of target modulation with drug mechanisms approved for other indications can identify high-confidence repurposing candidates, and was validated by genetically recapitulating established pharmacological treatments for diseases, known as ‘positive controls’ [14, 15]. Applying this framework to MD is particularly pertinent, where therapeutic mechanisms are thought to be complex [4]. Unlike prior MD drug target MR investigations [17, 18], we evaluate established antidepressant targets as positive controls to benchmark analyses and strengthen novel target prioritisation.

We applied an integrated genetic and pharmacological framework to systematically identify and prioritise MD targets within the druggable genome. We aimed to: i) identify targets using *cis*-MR and assess the genetic robustness of findings, ii) validate our approach using established MD target-compound pairs as positive controls, and iii) characterise repurposing opportunities by integrating genetic effects with drug mechanisms of action and clinical annotations of drug effects.

## Materials and methods

### Outcome and exposure data

#### GWAS summary statistics

For our outcome, we used summary statistics from the PGC MD phase 3 GWAS meta-analysis (525,197 cases and 3,362,335 controls) of European genetic ancestry (PGC MD-3) [16]. We derived exposure genetic instruments from 10 datasets measuring protein or transcript values in blood [20–22], cerebrospinal fluid (CSF) [23], or multiple brain regions [24, 25] (Supplementary Table 1). These genetic variants are quantitative trait loci (QTLs) strongly associated with changes in protein levels (pQTLs) or mRNA expression (eQTLs) in each tissue [10]. To maximise protein coverage and enable cross-platform validation [26], blood plasma proteomic data were derived from two datasets measured using different protein assay platforms: deCODE (SomaScan) and UK Biobank Pharma Proteomics Project (Olink; Supplementary Methods) [20, 21]. We analysed each QTL dataset separately.

#### Drug target data

We selected 3652 genes encoding target proteins for *cis*-MR analysis using hierarchical druggability assessments from Open Targets [27]. We included all targets with clinical trial evidence from ChEMBL [28]: approved drugs (phase IV, already ‘drugged’), advanced clinical development (phases II-III), and early clinical development (phase I). For targets with only experimental evidence (i.e. potentially druggable), we retained those within the top tier of experimental quality assessments (Supplementary Methods) to incorporate targets with promising druggability potential.

### Instrument selection

For each of the 10 QTL datasets, we applied the same instrument selection criteria. We selected common genetic variants as instruments with minor allele frequencies > 0.01, located within 200 kb of a target-encoding gene (*cis*-acting) [29], and with exposure GWAS p-value < 1 × 10^−6^. This p-value threshold corresponds to a minimum F-statistic of approximately 24 [30], exceeding the conventional threshold F ≥ 10 at which weak instrument bias is expected to be small [31]. Variants were clumped to linkage disequilibrium (LD) R² < 0.4 using a reference panel derived from 5000 UK Biobank participants of European genetic ancestry [32]. Remaining instrument correlations were accounted for by generalised least squares modelling. To mitigate potential horizontal pleiotropy, we applied heterogeneity-based pruning, removing variants with large leverage ( > 3 times the mean) or outlier statistics (chi-square >11) [19, 33]. We applied MR Steiger filtering to minimise reverse causation by assessing whether any variants had a greater effect on the outcome than on the exposure [34]. All instruments passed Steiger filtering, consistent with the minimal reverse causation expected in *cis*-MR, in which genetic effects of *cis*-variants on the corresponding molecular trait precede downstream effects on disease [10]. We annotated significant *cis*-MR targets ( ± 100 kb of gene boundaries) that overlapped with the major histocompatibility complex (MHC) region (chr6: 25–34 Mb). Given its complex LD structure, targets in the MHC should be interpreted with caution [35].

### Primary Mendelian randomisation (MR) analysis

We used purpose-built Python packages for *cis*-MR analyses (https://gitlab.com/cfinan/merit) [19]. We employed the Wald ratio estimator (MR-Wald) for targets instrumented by a single genetic variant, and the generalised least squares implementation of the inverse-variance weighted estimator (MR-IVW), allowing for correlated instruments [36] for targets with multiple variants. We implemented fixed-effects IVW for targets with <4 IVs and random-effects for ≥4 [37, 38]. A total of 3469 targets of the 3652 in the druggable genome had instrument(s) that passed our filtering criteria. We applied Bonferroni correction (p < 3.9 × 10^−6^) accounting for 12,794 MR-IVW/-Wald tests across 10 datasets spanning blood, brain and CSF. We used the total number of tests rather than the number of unique targets tested, as most targets (94.2%) were tested more than once across datasets (Supplementary Table 2).

### Sensitivity analyses and target annotation

#### MR-IVW robustness

We employed three robust methods, with different assumptions about instrument validity [39], across four models: MR-Egger with and without heterogeneity-based pruning [40], contamination mixture [41], and weighted median methods [42] (Supplementary Methods). We applied Bonferroni correction (p < 7.2 × 10^−5^) for 693 significant IVW estimates that we sought to test with robust MR. Robust MR estimates that were significant and directionally consistent with IVW for that target provided stronger evidence for the validity of our causal estimates [39].

#### External pQTL replication

We performed external replication of targets significant in our primary *cis*-MR analyses in independent QTL datasets, focusing on those with a matching molecular phenotype modality (i.e. pQTL) and tissue type (i.e., blood or brain) to our discovery datasets (Supplementary Methods and Supplementary Table 3). We defined successful replication as a significant MR-IVW/-Wald effect in the replication dataset that was directionally consistent with the discovery dataset effect estimate. The eQTLs were not tested for replication as they were derived from large-scale meta-analyses lacking comparably well-powered independent datasets.

#### Colocalisation

We performed colocalisation analyses for each significant MR-IVW/-Wald target to evaluate whether molecular traits (pQTL/eQTL) and MD share causal variants. We used the *coloc* R package and the *‘coloc.abf*’ function (Supplementary Methods) [43] with a posterior probability threshold of ≥ 0.8. Strong PPH4 evidence indicates a shared causal variant between the molecular trait and MD offering triangulation of evidence alongside *cis-*MR [29, 44]. Strong PPH3 evidence indicates distinct causal variants for each trait, suggesting potential violations of MR assumptions [29, 44].

#### Estimated therapeutic relevance

We estimated whether existing compounds targeting a *cis*-MR significant protein would act in a potentially beneficial direction on MD. We defined targets as MR-inferred ‘risk-increasing’ or ‘risk-decreasing’ based on whether higher expression or protein levels were associated with increased or decreased MD risk, respectively. We then integrated MR IVW/-Wald effect directions on MD with drug mechanisms of action data on each target from ChEMBL (Supplementary Methods) [28]. Using this integration, we classified the estimated therapeutic directional effect for each target-compound pair as one of three definitions. First, ‘beneficial’: target-compound mechanism aligns with reducing MD risk, i.e. an inhibitor for a risk-increasing target, or an activator for a risk-decreasing target. Second, ‘adverse’: target-compound mechanism is consistent with increasing MD risk, i.e. an activator for a risk-increasing target, or an inhibitor for a risk-decreasing target. Third, ‘unknown’: target-compound directional effect could not be classified (e.g., for mechanisms that do not specify inhibition or activation; Supplementary Table 4).

#### Known depression-related drug effects

We extracted depression-related therapeutic applications or side effects for compounds targeting *cis*-MR significant proteins from databases of drugs licensed in the UK and ChEMBL [28]. We classified drug effects using core depression terms (MD, depression, apathy, mood, anxiety, suicidality) and broader depression-relevant terms encompassing related psychiatric and somatic symptoms (Supplementary Methods). Depression-related clinical effects, including adverse effects, suggest biological relevance. Targets can be modulated by drugs with distinct mechanisms (activators or inhibitors), enabling the development of novel drugs with beneficial effects. Furthermore, as documented in databases of medications licensed in the UK, approved antidepressants have reported psychiatric side effects, including mood changes and anxiety. Thus, depression-related side effects do not necessarily preclude therapeutic potential.

### Positive control analysis

We identified positive controls using drug effects data from ChEMBL and databases of drugs licensed in the UK [28]. We selected targets of drugs with primary indications for depression and/or anxiety. We classified them as approved positive control targets (our primary positive controls, targeted by drugs in phase IV) and clinical trial positive control targets (our secondary positive controls, targeted by compounds in clinical trial phases I-III). We included anxiety due to high comorbidity, treatment and genetic overlap with MD, with genetic correlations approaching unity ( ~ 0.9) [45–47]. As a single compound can act on multiple targets, we also analysed our results at the compound level. We conducted enrichment analyses to assess whether our *cis*-MR approach identified known depression targets in brain and blood tissues separately (Supplementary Methods).

### Target prioritisation

**Fig. 1:**
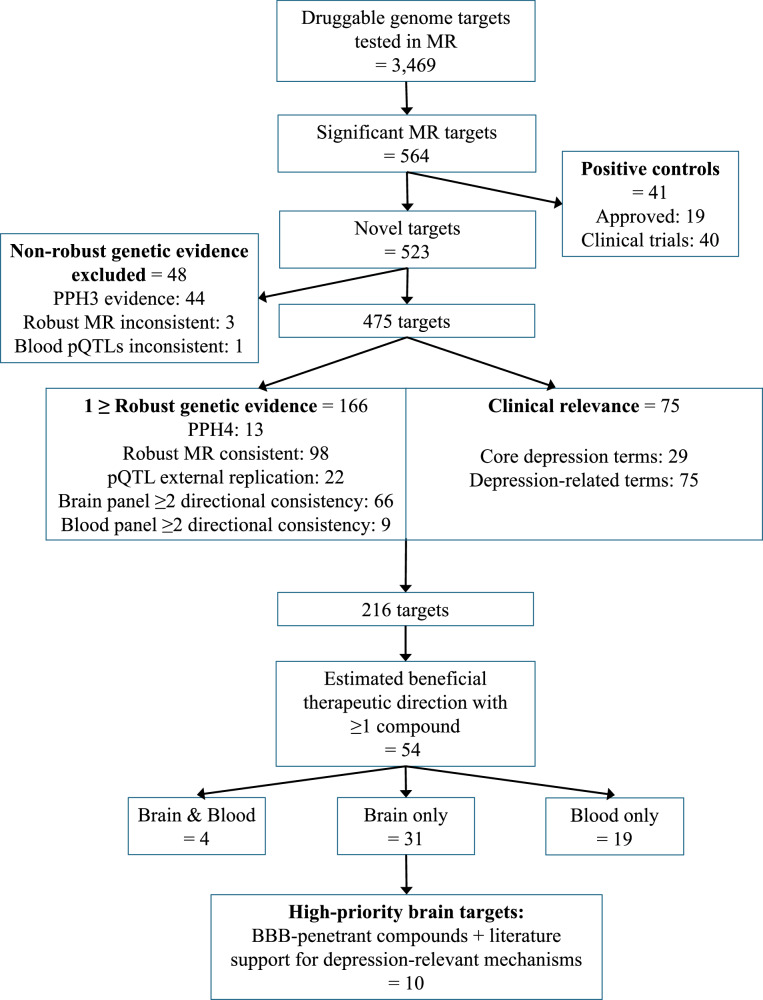
Novel target identification and prioritisation workflow. Flow diagram showing systematic identification of 10 high-priority novel drug targets for major depression (MD) from 3469 druggable genome targets tested in *cis*-Mendelian randomisation (MR; Bonferroni-corrected significance threshold p < 3.9 × 10^−6^). Positive controls were significant targets of compounds approved or in clinical trials for depression or anxiety. Novel targets were defined as significant targets not classified as positive controls. Novel targets with evidence suggesting a non-robust primary *cis*-MR estimate were excluded. Remaining targets meeting ≥1 robust genetic evidence criterion (n targets=166) or clinical relevance criterion (n = 75) were combined, with 25 targets meeting both criteria, yielding 216 unique targets. Targets were prioritised if ≥1 compound had a mechanism of action on the target estimated to reduce MD risk. Prioritised targets were categorised by tissue expression, with brain-only targets further evaluated against high-priority criteria.

We evaluated significant MR-IVW/-Wald targets by considering evidence for each target across assessments of genetic robustness and therapeutic potential. We applied a sequential selection approach to prioritise *novel* targets for MD, as illustrated in Fig. 1. We defined novel targets as those acted on by compounds not currently approved or tested in clinical trials for depression or anxiety (i.e., excluding positive controls). We aimed to identify potential repurposing drug candidates, defined as drugs approved for non-psychiatric conditions or tested in clinical trials for indications other than depression or anxiety.

First, we excluded novel targets with evidence suggesting a non-robust primary *cis*-MR estimate. Non-robust genetic evidence was defined as any instance of: i) colocalisation evidence for distinct causal variants, ii) a significant robust MR estimate that was directionally inconsistent with the primary MR-IVW estimate, or iii) directionally opposite *cis*-MR effects between blood pQTL discovery datasets using different protein assay platforms, indicating potential technical artefacts in protein measurement (Supplementary Methods).

Second, we retained targets that met either genetic robustness or clinical relevance criteria. We defined robust genetic evidence as i) PPH4 colocalisation evidence, ii) robust *cis*-MR estimates that aligned with the primary MR-IVW effect direction, iii) pQTL external replication, and iv) consistent directional *cis*-MR effects across ≥2 discovery datasets within the same tissue type (brain or blood). Not all targets had sufficient data or statistical power to be evaluated by each of these criteria, therefore we required ≥1 to be met or clinical relevance as an alternative criterion. We defined clinical relevance as targets acted on by repurposing compounds (developed for other conditions) with known depression-related drug effects.

Third, we restricted our prioritised targets to those with ≥1 compound that had a mechanism of action on the target acting in a direction that aligned with reducing MD risk (estimated beneficial therapeutic direction). Fourth, we then categorised prioritised targets by tissue expression (brain-only, blood-only or brain and blood). We focused on brain-only targets as potential high-priority candidates given the neurobiological basis of MD and that tissue-specific targets may produce fewer off-target effects than those ubiquitously expressed [48]. Finally, brain-only prioritised targets were defined as ‘high-priority’ as those meeting both of the following criteria through literature searches: i) blood-brain barrier permeability of estimated beneficial compounds, and ii) the target’s role in depression-relevant pathophysiological mechanisms. This included literature on the target function in relevant biological processes and evidence for antidepressant-like effects of beneficial compounds or target modulation in animal models or human studies.

### Protein-protein interaction analysis

**Fig. 2:**
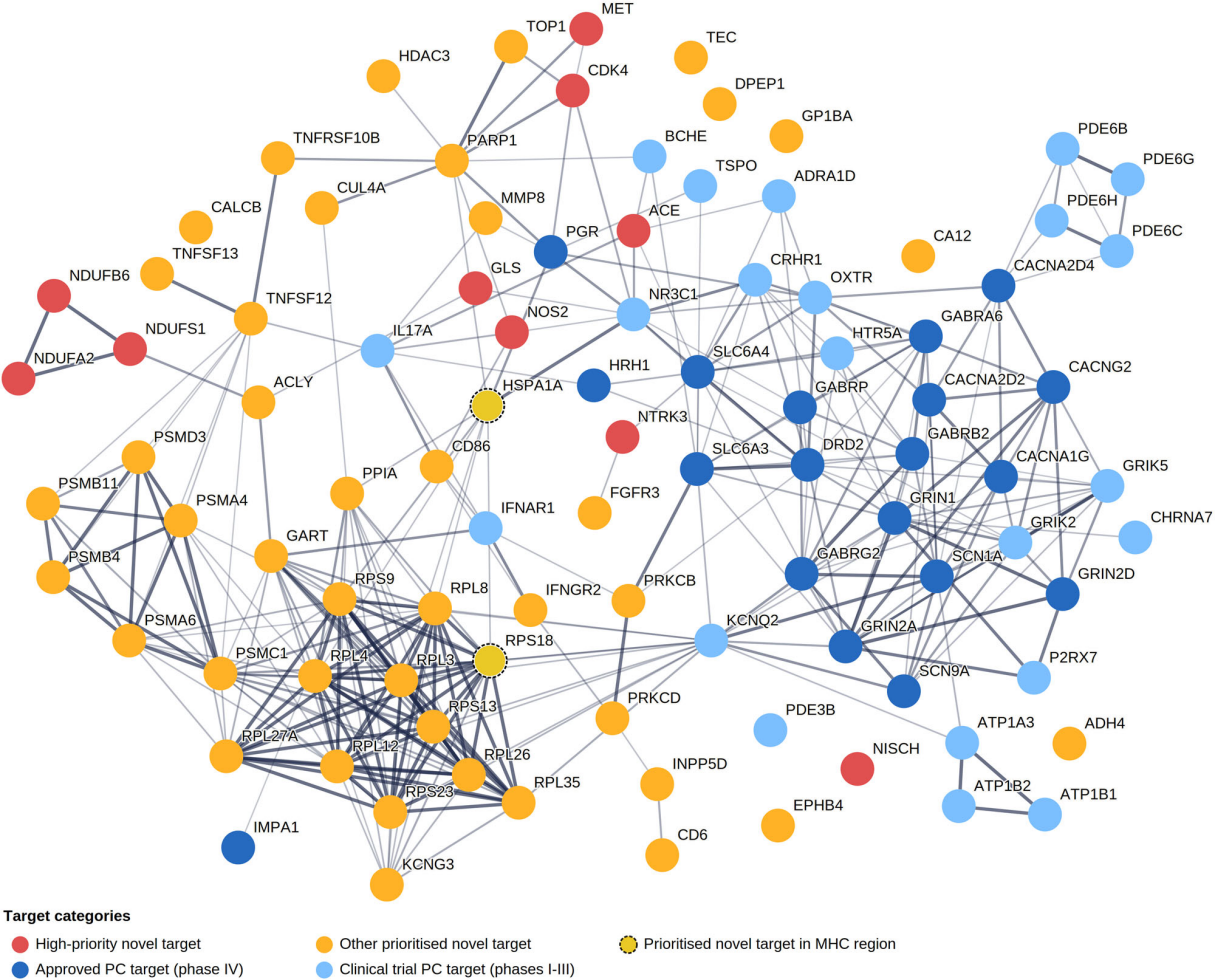
*Protein-protein interaction network of prioritised novel targets and positive control targets for major depression.* Network of 95 proteins queried in the STRING database. Proteins included 54 novel targets prioritised from *cis*-MR analysis (Fig. 1) and 41 positive control (PC) targets of compounds approved or in clinical trials for depression and/or anxiety. Each circle (node) represents a protein target, coloured by target category. Lines between targets (edges) represent known or predicted protein-protein interactions. Line thickness indicates interaction confidence based on a combined score integrating experimental, curated databases, text-mining, co-expression, conserved genomic proximity, evolutionary genomic co-occurrence and gene fusion evidence channels. Thicker lines indicate higher confidence with a minimum threshold of 0.4. The network was restricted to queried proteins only, with no additional interactors. Protein-protein interaction network enrichment was significant with 322 edges observed compared to 171 expected by chance for a random set of proteins of equal size and degree distribution (p < 1.0 × 10⁻¹⁶). MHC major histocompatibility complex.

To examine whether prioritised novel targets interact with positive control targets for depression and anxiety, we assessed protein-protein interactions (PPI) using STRING (v12.0) [49]. We queried 95 proteins using the Ensembl ID (GRCh38) of their encoding gene (54 prioritised novel targets and 41 positive control targets), retaining interactions with a combined evidence score > 0.4 (medium confidence threshold). The combined score integrates multiple evidence channels (Fig. 2). The PPI network was restricted to queried proteins only, with no additional interactors.

## Results

### Overview

Our primary *cis*-MR analysis identified 564 unique drug targets (of 3469 tested) that were significantly associated with MD across at least one of 10 QTL datasets measuring protein or transcript values in blood, brain, or CSF (see Supplementary Tables 5–14 for genetic results in each dataset). We evaluated significant targets by integrating evidence for genetic robustness and therapeutic potential (Fig. 1 and Supplementary Table 15). Target-level aggregated results from all analyses for each of the 564 significant targets are in Supplementary Table 16.

### Positive control analysis

Our *cis*-MR analysis demonstrated stronger genetic support for phase IV compounds for depression and anxiety (approved positive controls) than phase I-III compounds (clinical trial positive controls). At the drug level, 50/61 (82%) approved positive controls showed at least one significant *cis*-MR target effect on MD, compared to 86/169 (51%) for clinical trial positive control compounds (Supplementary Tables 17–19). Of the approved positive control drugs with significant targets, most had a mechanism of action on a target estimated to be beneficial for MD (i.e. target-compound mechanism aligns with reducing MD risk). Specifically, 27 drugs (54%) had exclusively beneficial target-compound mechanisms, while 13 drugs (26%) with multiple targets showed mixed effects (i.e. beneficial on some targets, adverse on others). Only 4 drugs (8%) exhibited exclusively adverse target-compound mechanisms (i.e. all target-compound mechanisms for that drug were estimated to increase MD risk), and 6 drugs (12%) had unknown directionality. The exclusively adverse classification of 4 drugs targeting DRD2, PGR, SCN1A, and SCN9A has different potential explanations (Supplementary Discussion). Notably, DRD2 is also targeted by positive control compounds with estimated beneficial mechanisms, illustrating that compounds with opposing pharmacological mechanisms on a target can be approved for the same indication. For compounds undergoing clinical trials, 29 (33.7%) showed beneficial target-compound mechanisms, 24 (27.9%) had mixed effects, 15 (17.4%) demonstrated adverse mechanisms, and 18 (20.9%) had unknown directionality. Table 1 presents results for targets of key positive controls with significant *cis*-MR effects and estimated therapeutic directions that align with their known clinical efficacy for MD and/or anxiety.

**Table 1:** Drug targets of positive control compounds for depression and anxiety with significant *cis*-MR effects and estimated beneficial therapeutic directions on major depression.

Target	Tissue (*cis*-MR effect direction)	Supporting genetic evidence	Estimated therapeutic MOA & drug MOA	Positive control compounds with aligned MOA	Primary indications
**Monoamine signalling**					
Sodium-dependent serotonin transporter: *SLC6A4*	eQTL blood (+)	Effect direction aligned with beneficial MOA therapeutic effects	Inhibition (-), 28 inhibitors/ antagonists (-)	21 SSRIs/SNRIs, TCAs, atypical antidepressants	Depression & anxiety
Sodium-dependent dopamine transporter: *SLC6A3*	eQTL cortex (+)	Effect direction aligned with beneficial MOA therapeutic effects	Inhibition (-), 10 inhibitors (-)	bupropion	Depression, ADHD (e.g. methylphenidate)
Dopamine receptor D2: *DRD2*	eQTLs (+): basal ganglia, hippocampus, spinal cord	Directional consistency, aligned with beneficial MOA therapeutic effects	Inhibition (-), 46 antagonists/ inhibitors (-)	amoxapine, quetiapine prochlorperazine	Depression, anxiety, bipolar disorder, schizophrenia
Histamine H1 receptor: *HRH1*	eQTL cortex (+), eQTL blood (+)	Directional consistency, aligned with beneficial MOA therapeutic effects	Inhibition (-), 62 antagonists/ inverse agonists (-)	hydroxyzine, dibenzepin	Depression & anxiety
**Glutamatergic signalling**					
NMDA receptor subunits: GRIN1 (+) GRIN2D (+) GRIN2A (-)	pQTL DLPFC (+), eQTL cerebellum (-), pQTL blood (+)	Largely consistent effect across subunits (+) aligned with beneficial MOA therapeutic effects	Inhibition/ activation (-/+), 21 negative allosteric modulator/antagonists/ blocker (-)	esketamine	Treatment-resistant depression
**GABA signalling**					
GABA(A) receptor subunits: *GABRA6* (-) *GABRB2* (-) *GABRG2* (-), *GABRP* (+)	eQTL cerebellum (-/+)	Largely consistent effect across subunits (-) aligned with beneficial MOA therapeutic effects of anxiolytics	Inhibition/ activation (-/+), 59 positive allosteric modulators/agonist (+)	benzodiazepines, brexanolone, meprobamate	Anxiety, insomnia, anxiety with depressive symptoms, postpartum depression
**Calcium ion channel modulators**					
Calcium channel subunits: *CACNG2* (+), *CACNA1G (+)*, *CACNA2D2* (+/-) *CACNA2D4* (-)	eQTLs (+/-): cerebellum, cortex, basal ganglia, hippocampus, blood	Largely consistent effect across subunits (+), robust MR support	Inhibition/ activation (-/+), modulators, blockers (-)	pregabalin	Generalised anxiety disorder, epilepsy, nerve pain
**Targets of compounds in clinical trials for depression/anxiety**					
Phosphodiesterases: *PDE6B* (+), *PDE6G* (+), *PDE6H* (+) *PDE3B* (+) *PDE6C* (-)	eQTL blood (+/-)	Largely consistent effect across subtypes (+) aligned with beneficial MOA	Inhibition/ activation (-/+), inhibitors (-)	9 inhibitors, e.g. pentoxifylline, dipyridamole	Cardiovascular (IV), depression (I-II)
Corticotropin -releasing factor receptor 1: *CRHR1*	eQTLs: cerebellum (-), cortex (+)	Cortex effect (+) aligned with beneficial MOA, robust MR support	Inhibition/ activation (-/+), 5 antagonists (-)	5 in clinical trial: e.g. verucerfont, emicerfont, pexacerfont	Depression & anxiety (I-II)

A positive (+) MR effect indicates higher expression or protein levels are associated with increased MD risk, suggesting beneficial compounds would have inhibitory (-) mechanisms of action (MOA) on the target; a negative (-) MR effect indicates higher expression or protein levels are associated with decreased MD risk, suggesting beneficial compounds would have activating (+) mechanisms of action. Consistent MR effect directions across related subunits/subtypes, concordance between tissues, and agreement between genetically estimated therapeutic direction and known clinical efficacy of established drug mechanisms strengthen the validity of our findings.

*ADHD* attention-deficit/hyperactivity disorder, *DLPFC* dorsolateral prefrontal cortex, *eQTL* expression quantitative trait loci, *MD* major depression, *MOA* mechanism of action, *MR* Mendelian randomisation, *pQTL* protein quantitative trait loci.

At the target-level, we found significant enrichment of targets of approved positive control compounds in brain tissues (OR = 1.97, 95% CI = 1.11–3.49, p = 0.018; Supplementary Table 20). Approved positive control targets had a 1.7-fold higher rate of significant *cis*-MR estimates (18.8%) in the brain compared to other targets (10.5%). Notably, the aforementioned compound-level rate (82%) was higher because most drugs act on multiple targets, and compounds required only one of those targets to be significant to be counted.

Targets of clinical trial positive controls showed similar brain-specific enrichment (OR = 1.54, 95% CI = 1.02–2.30, p = 0.036). Enrichment was not significant in blood tissues for either approved positive control targets (OR = 1.81, 95% CI = 0.75–4.35, p = 0.289) or clinical trial positive control targets (OR = 1.10, 95% CI = 0.60–2.03, p = 0.756). *Cis*-MR evidence for positive controls in brain tissues supports the biological validity of our approach, aligning with the neurobiological aetiology of MD and with established pharmacological interventions targeting neurotransmitter systems and neural circuitry [50].

### Prioritisation of novel targets for repurposing

We identified 523 drug targets beyond those currently used or explored in depression treatment. Following our systematic prioritisation framework (Fig. 1), 216 targets met criteria for genetic robustness evidence (166 targets) or clinical relevance (75 targets), with 25 meeting both. Of the 216 targets, 54 were prioritised due to being targeted by compounds with estimated beneficial therapeutic directions for MD (Supplementary Table 16). Tissue expression patterns of prioritised targets included four found in both brain and blood, 31 brain-only targets, and 19 blood-only targets. Of the 31 targets exhibiting brain-specific effects, 10 met our high-priority criteria with blood-brain barrier-penetrant compounds and supporting literature for depression-relevant mechanisms (Table 2 and Supplementary Table 21). Of note, 17 of 564 significant targets fell within the MHC region (Supplementary Table 16), all among novel rather than positive control targets. Two prioritised brain-only targets (*HSPA1A* and *RPS18*) are in the MHC region, though neither met high-priority criteria.

**Table 2:** High-priority brain targets of novel compounds with estimated beneficial therapeutic directions on major depression.

Target	Tissue (*cis*-MR effect direction)	Supporting genetic evidence	Estimated therapeutic MOA & available drug MOA	Beneficial compounds	Primary indications (depression-related side effects/ clinical trial phase)	Depression relevant mechanisms
Cyclin-dependent kinase 4: *CDK4*	eQTLs: basal ganglia (-), cerebellum (-), hippocampus (-), spinal cord (-), pQTL DLPFC (+)	Directional consistency eQTL brain, external replication pQTL	Mixed: inhibition/ activation (-/+), inhibitor (-)	Crosses BBB: abemaciclib	Breast cancer (fatigue, appetite, psychiatric)	Neuroplasticity, neural activity, neurogenesis
Nischarin Imidazoline receptor 1: *NISCH*	eQTLs: cerebellum (+), hippocampus (+), spinal cord (-)	Directional consistency cerebellum & hippocampus	Mixed: inhibition/ activation (-/+), agonist (+)	Crosses BBB: moxonidine, rilmenidine	Hypertension (insomnia, anxiety, depression, psychiatric)	Sympathetic regulation, stress response, target activation has animal model antidepressant-like effects
Nitric oxide synthase, inducible: *NOS2*	eQTLs: cortex (+), basal ganglia (+), cerebellum (+)	Directional consistency	Inhibition (-), inhibitor (-)	Clinical trial: tilarginine, KD7040, GW-274150; crosses BBB: BN-4	Clinical trial: cardiogenic shock (III), migraine (II)	Neuroinflammation, oxidative stress, target inhibition has animal model antidepressant-like effects
Glutaminase kidney isoform, mitochondrial: *GLS*	eQTLs: cortex (+), cerebellum (+)	Directional consistency	Inhibition (-), inhibitor (-)	Clinical trial: telaglenastat; crosses BBB: JHU-083	Clinical trial: cancer (II)	Neuroinflammation, glutamate signalling, target inhibition has animal model antidepressant-like effects
Neurotrophic receptor tyrosine kinase 3: *NTRK3*	eQTL cortex (+)	Colocalisation	Inhibition (-), inhibitor (-)	Crosses BBB: entrectinib, larotrectinib	Cancer (anxiety, depression, psychiatric, mood, appetite/weight, fatigue/sleep)	Neuroplasticity, monoamine signalling, HPA axis regulation
Angiotensin -converting enzyme: *ACE*	eQTL cortex (+)	MR effect direction aligns with drug MOA & reported mental health benefits	Inhibition (-), inhibitor (-)	Crosses BBB: e.g. captopril, fosinopril, lisinopril	Hypertension (anxiety, depression, mood, appetite/weight, insomnia/sleep)	Neuroinflammation, synaptic plasticity, serotonergic signalling, stress response, ACE inhibitors have antidepressant-like effects in animal models and clinical studies
Complex I NADH dehydrogenase subunits: *NDUFA2* (+) *NDUFB6* (+) *NDUFS1* (+)	eQTL cortex (+)	Consistent effect across subunits, robust MR support, reported mental health benefits	Inhibition (-), inhibitor (-)	Crosses BBB: metformin	Type 2 diabetes (appetite)	Mitochondrial function, neuroinflammation, metformin has antidepressant-like effects in animal models and human observational studies
Hepatocyte growth factor receptor: *MET*	eQTLs: basal ganglia (+), spinal cord (+)	Directional consistency	Inhibition (-), inhibitor (-)	Crosses BBB: capmatinib, cabozantinib	Cancer (appetite, fatigue, psychiatric)	Neuroplasticity, neurotrophic signalling, neuroinflammation

Prioritised targets with novel repurposing opportunities for MD, i.e. drugs already approved or > Phase I for other indications but not in clinical trial for depression/anxiety as a primary indication. A positive (+) MR effect indicates higher expression or protein levels are associated with increased MD risk, suggesting beneficial compounds would have inhibitory (-) mechanisms of action (MOA) on the target; a negative (-) MR effect indicates higher expression or protein levels are associated with decreased MD risk, suggesting beneficial compounds would have activating (+) mechanisms of action. Brain targets were prioritised based on: availability of compounds acting in the beneficial direction for at least one tissue, plus clinical relevance (depression-related side effects) or ≥ 1 robust genetic evidence (significant MR effects with directional consistency in multiple brain/nervous system tissues, robust MR support, colocalisation support or pQTL external replication), plus compounds with blood-brain barrier permeability and plausible depression-relevant biological mechanisms including pre-clinical evidence.

*BBB* blood-brain barrier, *DLPFC* dorsolateral prefrontal cortex, *eQTL* expression quantitative trait loci, *HPA* hypothalamic-pituitary-adrenal, *MD* major depression, *MOA* mechanism of action, *MR* Mendelian randomisation, *pQTL* protein quantitative trait loci.

### Protein-protein interaction analysis

We queried 54 prioritised novel targets and 41 positive control targets, identifying 322 interactions (Fig. 2**;** PPI enrichment p < 1.0 × 10⁻¹⁶; 171 expected; Supplementary Table 22). Much of this enrichment reflected dense clustering among ribosomal and proteasomal subunits (Supplementary Tables 22–23). We observed 36 interactions between prioritised novel and positive control targets. Five of ten high-priority targets (*ACE*, *CDK4*, *GLS*, *NOS2*, *NTRK3*) showed direct connections with positive control targets, particularly *NR3C1* (encoding the glucocorticoid receptor) and *SLC6A4* (serotonin transporter). Both MHC region targets also interacted with positive control targets through experimental evidence (*HSPA1A* with *NR3C1* and *PGR*; *RPS18* with *KCNQ2*), suggesting potential relevance of this region to depression. Excluding ribosomal subunit interactions, most novel-to-positive control target interactions were supported primarily by text-mining evidence (19 of 25; 76%). *GLS*-*NR3C1* was a notable exception with predominantly experimental support. Among high-priority targets, *CDK4-MET* interaction was also supported by experimental evidence.

## Discussion

We identified novel drug targets and immediate repurposing opportunities for MD by triangulating genetic evidence and clinical data. Our integrated framework strengthens causal inference in target identification, guiding repurposing. The target-compound pairs we report span MD drug development stages. These include long-standing antidepressants, recently approved treatments, repurposing compounds in MD clinical trials or with early clinical evidence not yet formally tested for MD treatment, and candidates with strong preclinical evidence.

### Genetic validation through established targets and mechanisms

Our findings suggest that genetic evidence informs clinical success in MD drug development, with 82% of phase IV positive control drugs having significant targets, versus 51% for earlier phases I-III compounds. We corroborate broader findings that compounds supported by genetic data for complex traits are more likely to be approved [7]. Additionally, targets of positive control compounds showed brain enrichment, validating our focus on brain targets for repurposing.

For monoaminergic targets of approved MD treatments, *SLC6A4*, *SLC6A3*, and *DRD2* showed risk-increasing effects, aligning with the established therapeutic inhibitory mechanisms of SSRIs, bupropion, and quetiapine, respectively. Genetic support for DRD2 inhibition was consistent across brain regions, including the basal ganglia, reflecting its role in reward processing [51]. We thus provide directional insight into PGC MD-3 findings, which identified *DRD2* as the strongest gene-based association and antipsychotic compound enrichment [16]. Beyond monoaminergic signalling, we also found genetic support for anxiety medications, reflecting high comorbidity and genetic overlap between these disorders [45–47].

Positive control compounds with multiple targets showed both exclusively beneficial and mixed therapeutic effects. For esketamine, risk-increasing effects for *GRIN1* and *GRIN2D* aligned with the therapeutic benefit of inhibition, but were adverse for risk-decreasing *GRIN2A*, consistent with mixed therapeutic and side effect profiles for different NMDA receptor subunits [52, 53]. Similar patterns were observed among GABAA receptor subunits targeted by anxiolytic benzodiazepines. Subunit-specific effects suggest which receptor subunits may drive therapeutic benefits versus contribute to side effects [52–54]. Our findings could potentially inform the development of more selective compounds with improved efficacy-to-side-effect profiles for MD.

### Cardiovascular and metabolic repurposing compounds

Our genetic evidence supports repurposing multiple cardiovascular and metabolic system drugs from different stages in the repurposing pipeline. For pentoxifylline, a PDE inhibitor approved for vascular disease and tested in depression clinical trials [55–58], inhibition aligned with four risk-increasing PDE subtypes but was adverse for risk-decreasing *PDE6C*. Prioritising specific PDE subtypes for MD treatment may improve efficacy [59]. A meta-analysis of Phase II trials found that adjunctive pentoxifylline significantly improved depressive symptoms [60]. Among compounds with preliminary clinical evidence yet to be formally evaluated in MD clinical trials, *ACE* showed a risk-increasing effect in cortical tissue. Blood-brain barrier-penetrant antihypertensive ACE inhibitors, such as captopril, have demonstrated antidepressant-like effects in animal models and mental health benefits as secondary outcomes in clinical trials [61, 62]. Proposed mechanisms include reducing oxidative stress, neuroinflammation and stress-induced hypothalamic-pituitary-adrenal (HPA) axis activation, while enhancing serotonergic signalling [63], consistent with ACE interactions with the serotonin transporter and glucocorticoid receptor in PPI analysis. Both PDE inhibitors and ACE inhibitors are widely used and well tolerated in cardiovascular medicine, further underscoring their potential for repurposing.

We identified risk-increasing effects for Complex I subunits in cortical tissue (*NDUFA2*, *NDUFB6*, *NDUFS1*), aligning with metformin’s inhibitory mechanism on these targets, a type 2 diabetes medication. This complements the identification of *NDUFAF3*, another metformin target-encoding gene, as a novel MD-associated gene [64]. Metformin exhibits antidepressant and anxiolytic-like effects in animal models [65, 66] and is associated with decreased risk of depression in observational case-control studies [67]. Whilst one phase III trial demonstrated metformin’s efficacy in treatment-resistant bipolar depression [68], prioritising metformin for clinical trials with MD as a primary outcome is warranted.

Preclinical evidence supports the potential of antihypertensive imidazoline I_1_ agonists for MD (Supplementary Discussion). We found mixed effects of *NISCH* (encoding Nischarin, the proposed I_1_ receptor) across brain regions. Risk-increasing effects align with postmortem findings of elevated Nischarin in MD prefrontal cortex [69]. Endogenous I_1_ agonist agmatine shows compensatory increased brain levels during stress with antidepressant-like effects in rodents [70–72], and SSRI efficacy may be partially mediated by I₁ activation [73, 74].

### Cancer repurposing compounds

Among our prioritised novel targets were three proteins inhibited by blood-brain barrier-penetrant cancer drugs, with plausible roles in neural function. *CDK4* showed mixed directional effects across five brain regions, reflecting diverse neural functions regulating neurogenesis [75], neuronal activity [76] and synaptic plasticity [77]. *NTRK3* encodes the receptor for neurotrophin-3, which modulates monoaminergic transmission, brain-derived neurotrophic factor expression and HPA axis function [78]. We corroborate the PGC MD-3 identification of *NTRK3* as a high-confidence MD gene [16] whilst providing novel directional insights by identifying inhibitors to counteract the risk-increasing effect of NTRK3. *MET* encodes the MET hepatocyte growth factor receptor, involved in regulating neuroplasticity, neurotrophic pathways, and inflammation [79]. Approved inhibitors for all three targets exhibit psychiatric side effects, supporting their neuropsychiatric relevance [80–82]. However, the CDK4-selective inhibitor abemaciclib demonstrates the potential for an improved nervous system safety profile when repurposing cancer drugs for MD, with fewer psychiatric side effects than less selective alternatives [82]. Further investigations of CDK4, NTRK3 and MET inhibitors in MD are warranted, such as dose-ranging studies or selective compound development to optimise efficacy while minimising side effects.

### Early-stage drug development compounds

We identified two enzyme targets inhibited by experimental compounds yet to be clinically approved for any indication or tested in MD clinical trials, representing opportunities beyond the repurposing of approved medications. Inhibiting GLS (glutaminase) offers a mechanistically distinct glutamatergic modulation compared to approved depression compounds. While NMDA antagonists block glutamate receptor signalling, glutaminase inhibition reduces glutamate production in activated microglia, directly limiting excitotoxicity and neuroinflammation [83, 84]. The novel blood-brain barrier-penetrant glutaminase inhibitor JHU-083 demonstrates antidepressant-like effects in animal models while minimising peripheral side effects as a pro-drug [83, 84]. More recently, glutaminase inhibitors selectively targeting activated microglia attenuate depressive-like behaviours following chronic stress [85]. Similarly, inhibition of inducible nitric oxide synthase (iNOS, encoded by *NOS2*) targets glial-induced neuroinflammation [86, 87]. Unlike other NOS isoforms, iNOS offers temporal and cell-type-specificity to reduce neuroinflammation without affecting normal NO signalling [86, 87]. Selective iNOS inhibition in rodents exhibits antidepressant-like effects [88, 89] and attenuates stress-induced anxiogenic effects [90]. Our PPI findings with the glucocorticoid receptor align with antidepressant-like effects of GLS and iNOS inhibition during stress potentially involving glucocorticoid pathways. GLS inhibition may attenuate glucocorticoid receptor-driven excess glutamate release [91], while iNOS inhibition may limit stress-induced nitric oxide production that impairs glucocorticoid receptor function [92]. To our knowledge, we provide the first human genomic evidence for these preclinical compounds, highlighting the potential of these compounds as novel MD therapeutics targeting neuroinflammatory and stress-induced pathways.

### Strengths, limitations and future directions

Compared with prior *cis*-MR analyses of MD scanning the druggable genome, which identified 7 and 23 significant targets, most of our 54 prioritised targets were not identified in either prior study [17, 18]. This likely reflects our use of a larger MD GWAS and broader coverage of QTL datasets. Regarding novel targets, *PSMB4* was prioritised across all three studies with concordant risk-increasing effect directions [17, 18]. Some of our positive control targets were also significant in prior studies [17, 18]. This includes *P2RX7*, identified in all three studies with concordant risk-decreasing directions. Prior studies did not map *P2RX7* to compounds, whereas we classified existing antagonists as having an estimated adverse therapeutic effect [17, 18]. *CRHR1* and *GRIK2* were also identified by Li et al. [18] as potential novel targets, though we classified these as positive controls given existing clinical trial evidence in depression [18]. Several overlapping significant targets did not meet our robustness and therapeutic potential criteria for prioritisation [17, 18].

Whilst we prioritised drugged targets for rapid translation and included high-quality experimental targets beyond prior studies [17, 18], other pQTL/eQTLs may become therapeutically viable [9]. Lifelong genetic effects may differ from acute pharmacological interventions [93]. QTL data availability constrains which tissues and cell types can be tested, which may not capture the most therapeutically relevant contexts [10, 29]. Furthermore, c*is*-MR examines the effects of individual proteins, however, proteins function within complex pathways [94], challenging estimation of therapeutic effects from single-target modulation. Polypharmacological effects can trigger broader network responses and compensatory mechanisms [94, 95]. While *cis*-MR reduces the risk of horizontal pleiotropy and we applied robust MR estimators, it cannot be entirely ruled out [10]. Only 11% of targets had sufficient power for colocalisation, which requires strong *cis*-region GWAS associations in both traits [96]. Our analysis of European-associated ancestry data may limit generalisability to other populations [97].

Blood-brain barrier permeability annotations were derived from the literature and likely vary in evidence quality, with some classifications based on limited experimental data or predicted chemical properties rather than direct measurements. PPI networks from literature-curated databases are subject to bias, with well-studied proteins, including drug targets, showing inflated connectivity that may not reflect biological relevance [98]. Given this bias as highlighted by the predominance of text-mining evidence among our observed interactions, we incorporated PPI findings as descriptive annotations rather than as prioritisation criteria.

MD heterogeneity challenges the translation of genetically supported targets and compounds into treatments. Despite genetic evidence from our study and previous work [18], CRHR1 antagonists targeting stress dysregulation have failed in depression clinical trials [99]. Similarly, anti-inflammatory drug clinical trials have largely been unsuccessful [100], potentially due to ‘one-size fits all’ approaches that obscure therapeutic effects [100, 101]. Future work could explore genetically supported compounds through patient stratification, matching treatments to MD subtypes [100, 101].

### Conclusions

Our genetically informed approach corroborated established MD targets while identifying novel therapeutic opportunities beyond traditional monoaminergic mechanisms. Several novel targets have approved drugs for other indications with established safety profiles and blood-brain barrier penetration, offering opportunities for immediate clinical evaluation.

## Supplementary information

Supplementary Materials

Supplementary Tables

## Data Availability

GWAS summary statistics for MD are available from the Psychiatric Genomics Consortium at https://pgc.unc.edu/for-researchers/download-results/. GWAS summary statistics including 23andMe data require an approved application through 23andMe available to qualified researchers under an agreement with 23andMe that protects the privacy of the 23andMe participants (visit https://research.23andme.com/dataset-access/). UK Biobank data are available through application at https://www.ukbiobank.ac.uk/enable-your-research/apply-for-access/. Open Targets tractability data (version dated 2024-05-23) can be downloaded from http://ftp.ebi.ac.uk/pub/databases/opentargets/platform/latest/input/target/tractability/. QTL datasets are available from: Blood plasma pQTL data: deCODE (https://www.decode.com/summarydata/), UKB-PPP (https://www.synapse.org/#!Synapse:syn51364943/), INTERVAL (http://www.phpc.cam.ac.uk/ceu/proteins/), and Gudjonsson (https://www.ebi.ac.uk/gwas/publications/35078996). Brain pQTL data: ROSMAP and Banner (https://www.synapse.org/#!Synapse:syn24172458). CSF pQTL data: Yang (https://dss.niagads.org/datasets/ng00102/). Blood eQTL data: eQTLGen (https://www.eqtlgen.org/). Brain eQTL data: MetaBrain (https://www.metabrain.nl/). Compound mechanism of action and drug effect data were obtained from ChEMBL (v33; https://www.ebi.ac.uk/chembl/). C*is*-MR results and genetic instruments for all targets tested, including non-significant results, are available on Zenodo (10.5281/zenodo.19135141).

## Code availability

*Cis*-Mendelian randomisation and colocalisation analyses were performed using purpose-built Python (v3.9) packages (Mendelian Randomisation for Identifying Targets [MeRIT], v0.3.3a0; https://gitlab.com/cfinan/merit), which incorporates the *coloc* R package for colocalisation (https://github.com/chr1swallace/coloc). Estimated therapeutic relevance and known depression-related drug effects were derived using *bio-misc* (v0.2.0a0; https://gitlab.com/cfinan/bio-misc). GWAS summary statistics were processed and standardised using *gwas-norm* (https://gitlab.com/cfinan/gwas-norm). Druggable genome targets were defined using Open Targets tractability assessments (https://github.com/chembl/tractability_pipeline_v2; https://platform-docs.opentargets.org/target/tractability). Protein-protein interaction network analysis was conducted using STRING (v12.0; https://string-db.org).
