# Supplementary material for: An integrative mendelian randomisation and drug mechanism framework for target prioritisation and therapeutic repurposing in major depression
Source: Transl Psychiatry. 2026 Jun 2;16:392. doi: 10.1038/s41398-026-04137-9 (PMC13443190; doi:10.1038/s41398-026-04137-9)
Supplement: Supplementary file 1 [file 41398_2026_4137_MOESM1_ESM.docx]

**Supplementary materials for: “An integrative Mendelian randomisation and drug mechanism framework for target prioritisation and therapeutic repurposing in major depression”**

**Table of Contents**

**Supplementary Methods	1**

GWAS summary statistics	1

Drug target selection and druggability assessment	2

Instrument selection	3

MR-IVW robustness	3

External pQTL replication	4

Colocalisation	4

Estimated therapeutic relevance	4

Known depression-related drug effects	5

Positive control (PC) analysis	5

**Supplementary Discussion	6**

Interpretation of adverse-classified positive control compounds	6

NISCH	7

**References	7**

## Supplementary Methods

### GWAS summary statistics

All analyses were performed using the human reference genome GRCh38 reference assembly, and GWAS data were harmonised using the Python package *gwas-norm* [1]. For proteomic data, where possible, we selected GWAS of protein values measured using different assay platforms. Due to limited data availability in the brain and CSF, this was only feasible for blood plasma. We selected the two most well-powered blood plasma GWAS of protein values measured using two commonly used high-throughput proteomic platforms (deCODE using SomaScan and UK Biobank Pharma Proteomics Project [UKB-PPP] using Olink) [2, 3]. While these platforms show only modest correlations in their protein measurements due to different binding mechanisms and protein measure normalisation approaches, using both provides complementary information [4]. First, it enables testing non-overlapping proteins unique to each platform, improving overall protein coverage. Second, for overlapping proteins measured on both platforms, concordant findings provide stronger evidence of reliable protein quantification [4]. We deprioritised blood pQTL targets that showed significant but directionally opposite MR-IVW/-Wald effect estimates between our deCODE and UKB-PPP. These QTL datasets measure the same molecular trait (proteins) in the same tissue type (blood plasma) but using different protein assay platforms. Thus, finding opposite directions between these datasets could indicate potential technical artefacts in protein measurement. We did not penalise targets for directional inconsistencies between other tissues or molecular trait modalities, as such differences might reflect genuine biological variation across tissues or in protein versus gene expression rather than technical artefacts.

### Drug target selection and druggability assessment

We used Open Targets tractability data to define the druggable genome (version dated 2024-05-23). This framework assessed the druggable potential (or ‘druggability’) of 18,868 protein-coding genes based on their amenability to four drug modalities. This included small molecules, monoclonal antibodies [5], an emerging therapeutic approach called proteolysis targeting chimeras (PROTACs) [6], and ‘other modalities’ (e.g. biologics, oligonucleotides).

Open Targets incorporated clinical trial evidence from ChEMBL for all four modality groups. ChEMBL targets of drugs and/or clinical trial compounds encompassed both single proteins or multiple proteins functioning as a protein complex or protein family [7]. ChEMBL may collapse such multi-subunit complexes acted on by a compound into single target entries [7]. However, as Open Targets uses Ensembl gene identifiers to define targets, each gene encoding a complex subunit is treated independently as a separate target in our analysis. ChEMBL includes primary drug targets (i.e. proteins directly responsible for therapeutic effects). This is a stricter definition than those used in other databases, such as DrugBank, which include broader definitions with secondary targets [8, 9]. A more conservative approach aligns with our objective of identifying targets with the strongest evidence and thus the most promising for repurposing.

For experimental druggability assessments, this included data for small molecules, antibodies, and PROTACs. Experimental data were drawn from various databases incorporated by Open Targets, including UniProt, Human Protein Atlas, Protein Data Bank, and DrugEBIlity, evaluating factors such as protein structure, cellular location, and previous research evidence (see further details below). Our approach yielded 3,652 genes encoding drug target proteins, comprising 1,452 targets with clinical trial evidence (935 phase IV, 489 phase II-III, 28 phase I) and 2,200 with high-quality experimental evidence.

We retained targets meeting at least one tractability assessment across the four modalities, each with hierarchical bucket scores. The ‘clinical precedence’ category comprised bucket 1 (targets of phase IV drugs), bucket 2 (targets of compounds in advanced clinical trials, phases II-III), and bucket 3 (targets of compounds in early clinical trials, phase I). For experimental evidence, we included targets with the highest quality assessments (bucket 4) within each modality. Targets with experimental evidence for small-molecule binding required the highest ‘discovery precedence’ category evidence score of 1. This included targets with both structural ligand evidence (co-crystallised structure with a small molecule) and compound quality evidence (high-quality ligands meeting drug-like criteria). Targets with evidence for antibody-based therapy required a ‘predicted tractable high confidence evidence’ score of 1, with a high confidence accessible subcellular location from both UniProt and Gene Ontology classifications. PROTAC targets required literature precedence (documented in PROTAC development publications) combined with high confidence locations for the PROTAC approach (cytoplasm/nucleus location [scores 1-2]). Further details on tractability bucket criteria are available in the Open Targets tractability pipeline documentation at https://github.com/chembl/tractability_pipeline_v2 and https://platform-docs.opentargets.org/target/tractability.

### Instrument selection

We selected genetic variants as instruments at p < 1×10⁻⁶, a slightly less stringent threshold than the genome-wide 5×10⁻⁸. Less stringent thresholds are commonly used in *cis*-MR to increase instrument availability, which improves performance and enables pleiotropy-robust MR analyses [10–12]. In two-sample MR, any potential weak instrument bias attenuates towards the null rather than inflating false positives [10, 13, 14].

### MR-IVW robustness

For generalised least squares MR-Egger, which allows for correlated instruments, we applied an LD R-squared threshold of 0.4 using the same random sample of 5,000 UKB participants as our primary analysis to generate correlation matrices. For contamination mixture and median models, we used a more stringent clumping threshold of 0.01. We restricted robust MR estimates to targets with at least 3 IVs, as this is the minimum number of IVs required for both MR-Egger and median-based methods. For MR-Egger, we used a random-effects model [15]. Targets with significant but inconsistent directional effects across IVW and robust estimators were deprioritised.

### External pQTL replication

We used the same parameters for replication analyses as those applied in our primary *cis*-MR analysis. For blood pQTL targets significant in our discovery datasets (deCODE and UK Biobank), we tested for replication in two independent blood pQTL datasets (INTERVAL and Gudjonsson) [16, 17]. Both replication datasets use SomaScan platforms, providing within-platform replication for deCODE results, while also providing cross-platform replication for the UKB-PPP Olink measurements. Brain pQTL targets identified in ROSMAP were tested for replication in the Banner pQTL dataset, as these datasets share the same protein assay platform and brain regions [18]. We note there was no overlap between targets identified in the discovery blood pQTL and brain pQTL datasets. We therefore applied tissue-specific Bonferroni corrections, with separate thresholds for blood and brain based on the number of significant targets carried forward from their respective discovery datasets to replication datasets. For blood pQTLs, we accounted for targets tested more than once in blood replication datasets by correcting for the total number of tests (Supplementary Table 3).

### Colocalisation

We set the Bayesian prior probability that a variant is associated with either the molecular trait (p1) or MD (p2) individually as p1 = p2 = 1×10⁻⁴ and a lower prior probability for shared associations with both traits (p12 = 1×10⁻⁵), consistent with the recommended threshold for eQTL studies [19]. We set the genomic window size to match that of our *cis*-MR analyses [20]. Strong PPH3 evidence indicates distinct causal variants for each trait, suggesting potential violations of MR assumptions due to, for example, linkage disequilibrium, or pleiotropic pathways through neighbouring genes [20, 21]. For each target, we assessed the statistical power of our colocalisation analysis by examining the sum of posterior probabilities for hypotheses 3 and 4 (PPH3 + PPH4). A low combined probability for these hypotheses indicates limited statistical power to detect colocalisation [22, 23]. We therefore established a power threshold where the sum of PPH3 and PPH4 needed to reach at least 0.8 to consider the analysis sufficiently powered [23]. This threshold helped us distinguish between true negative results and those arising from insufficient statistical power.

### Estimated therapeutic relevance

We estimated the therapeutic relevance of each compound for MD by integrating drug mechanisms of action data from ChEMBL (v33) [7] with our primary MR results using the *bm-therapeutic-direction* function in the *bio-misc* package (v0.2.0a0) (https://gitlab.com/cfinan/bio-misc). We estimated whether existing compounds targeting that protein would act in a potentially beneficial direction based on the MR-IVW/-Wald effect direction. We classified drug mechanisms of action as either activatory or inhibitory based on their mechanism of action on target proteins. Activatory mechanisms included agonists, partial agonists, activators, positive allosteric modulators, and openers. Inhibitory mechanisms comprised antagonists, allosteric antagonists, blockers, inverse agonists, negative allosteric modulators, antisense inhibitors, degraders, and inhibitors (Supplementary Table 4).

### Known depression-related drug effects

Drug effects data were extracted using the *bm-search-drug-effects* function from the *bio-misc* package. This function queries databases of drugs licensed in the UK (primary therapeutic indications, side effects, cautions, and contraindications) and ChEMBL (v33) (primary compound indications in clinical trial phases I-IV) [7]. Depression-related drug effects could indicate: (1) for primary therapeutic indications: the compound paired to the target is relevant for the treatment of depression-related symptoms, ranging from early-stage clinical trials to approved medications (phases I-IV); (2) for side effects, cautions, or contraindications: depression-related effects are reported for the compound. We developed two distinct sets of terms to classify drug effects based on diagnostic criteria: core depression terms and terms more broadly related to depression, including psychiatric and somatic symptoms. Core depression terms captured primary depression-associated drug effects, including major depressive disorder, depression, apathy, mood, anxiety, and suicidality. The related depression terms encompass broader psychiatric and somatic symptoms associated with depression, including changes in activity, appetite, social behaviour, concentration, weight, restlessness, and sexual interest (i.e. loss of interest or pleasure). Broader but related terms that captured the effects of psychiatric medication included ‘psychiatric’, ‘abnormal thinking’, ‘negative symptoms’ (of psychosis and schizophrenia that overlap with depression) and ‘feeling abnormal’.

### Positive control (PC) analysis

We were able to test in *cis*-MR targets for 61 approved compounds and 169 clinical trial compounds. Compounds approved for one indication (e.g. MD) but in clinical trials for another (e.g. anxiety) were classified only in the approved category. At the target level, we tested 80 approved PC targets and 205 clinical PC targets. We note that 76 approved PC targets overlapped with clinical trial PC targets, reflecting that targets of approved drugs are also targets of other compounds tested in clinical trial phases I-III.

We separately compared significance rates in brain and blood tissues of 1) approved PC targets versus all other targets, and 2) clinical trial PC targets versus all other non-PC targets. We constructed contingency tables for brain and blood tissues separately, counting targets with significant and non-significant *cis*-MR estimates in each category. Comparisons were restricted to brain and blood due to the limited number of targets found in CSF. Odds ratios (OR) quantified enrichment, calculated as: (PC target significant/other target significant)/(PC non-significant target/other non-significant target). An odds ratio (OR) > 1 indicated higher odds of PC targets among significant results. Statistical significance was tested using chi-square tests, with Yates' correction applied when expected cell counts were < 5, and a p-value < 0.05 considered statistically significant.

## Supplementary Discussion

### Interpretation of adverse-classified positive control compounds

For four approved positive control compounds, all target-compound mechanisms were estimated to be adverse for MD. The exclusively adverse classification of these compounds targeting DRD2, PGR, SCN1A, and SCN9A has different potential explanations.

Compounds with opposing pharmacological mechanisms on a target can be approved for the same indication. DRD2 showed risk-increasing effects on MD, aligning with inhibition estimated as being beneficial. Both DRD2 antagonists (quetiapine; beneficial) and DRD2 partial agonists (aripiprazole, brexpiprazole; adverse) are approved as adjunctive therapy for MD. Both mechanisms can be clinically effective but may work better for some patients than others, reflecting heterogeneity in MD. Clinicians tend to prescribe these drugs for different symptom profiles [24]. Our genetic findings may therefore capture effects more relevant to certain symptom presentations.

The therapeutic effects of some compounds may be specific to depression subtypes that differ in aetiology. PGR showed risk-increasing effects on MD, suggesting that inhibitory compounds would be beneficial. Progesterone (PGR agonist; adverse) is licensed for postnatal depression, consistent with the abrupt fall in progesterone after childbirth being implicated in postpartum mood symptoms. Outside the postpartum context, progesterone is associated with adverse mood effects in some individuals [25], consistent with our findings. Conversely, mifepristone (PGR antagonist; beneficial) has been explored in Phase 3 trials for MD with psychotic features [26], and Phase 2 for MD following childhood trauma [27]. Our finding that PGR is a risk-increasing target for broad MD suggests PGR antagonism may warrant investigation in MD beyond psychotic or trauma subtypes.

The therapeutic effects of lamotrigine on mood may arise through other targets not identified in our analysis. We found risk-increasing effects for sodium channel subunits (SCN1A and SCN9A) on MD, suggesting that activation of these targets would be beneficial. Lamotrigine (SCN1A/SCN9A blocker; adverse) is used to treat depression, primarily in bipolar disorder. Only blockers of SCN1A and SCN9A currently exist. Lamotrigine's therapeutic effects on low mood may therefore arise through effects on other sodium channel subunits that did not reach significance in our analysis [28, 29]. Alternatively, effects may arise through secondary targets and downstream modulation of glutamatergic and monoaminergic signalling [29].

Notably, we found such an exclusively adverse classification for only a very small minority of positive control compounds. Such findings could arise by chance or from the erroneous estimation of the effect sign in *cis*-MR analyses (e.g., sign flipping). Emerging evidence suggests that such sign flipping can occur with gene expression data [30].

### *NISCH*

We found mixed effects of *NISCH* (encoding Nischarin, the proposed I_1_ receptor) across brain regions, likely reflecting context-dependent roles in neuroinflammation and neural plasticity [31, 32]. I_1_ agonist agmatine may be an endogenous antidepressant [33], with compensatory increased brain levels during stress and antidepressant-like effects in rodents [34–36] alongside decreased *NISCH* expression in the amygdala [37]. SSRI efficacy may be partially mediated by I_1_ activation [38, 39]. Consistent with our risk-increasing brain effects, Nischarin was elevated in postmortem prefrontal cortex of MD patients but lower in those taking antidepressants [40]. These findings establish Nischarin as a relevant MD target through potential involvement in stress-depression pathways and antidepressant action.

## References

1.	Schmidt AF, Bourfiss M, Alasiri A, Puyol-Anton E, Chopade S, van Vugt M, et al. Druggable proteins influencing cardiac structure and function: Implications for heart failure therapies and cancer cardiotoxicity. Sci Adv. 2023;9:eadd4984.

2.	Ferkingstad E, Sulem P, Atlason BA, Sveinbjornsson G, Magnusson MI, Styrmisdottir EL, et al. Large-scale integration of the plasma proteome with genetics and disease. Nat Genet. 2021;53:1712–1721.

3.	Sun BB, Chiou J, Traylor M, Benner C, Hsu Y-H, Richardson TG, et al. Plasma proteomic associations with genetics and health in the UK Biobank. Nature. 2023;622:329–338.

4.	Eldjarn GH, Ferkingstad E, Lund SH, Helgason H, Magnusson OT, Gunnarsdottir K, et al. Large-scale plasma proteomics comparisons through genetics and disease associations. Nature. 2023;622:348–358.

5.	Brown KK, Hann MM, Lakdawala AS, Santos R, Thomas PJ, Todd K. Approaches to target tractability assessment - a practical perspective. Medchemcomm. 2018;9:606–613.

6.	Schneider M, Radoux CJ, Hercules A, Ochoa D, Dunham I, Zalmas L-P, et al. The PROTACtable genome. Nat Rev Drug Discov. 2021;20:789–797.

7.	Mendez D, Gaulton A, Bento AP, Chambers J, De Veij M, Félix E, et al. ChEMBL: towards direct deposition of bioassay data. Nucleic Acids Res. 2019;47:D930–D940.

8.	Sadler MC, Auwerx C, Deelen P, Kutalik Z. Multi-layered genetic approaches to identify approved drug targets. Cell Genom. 2023;3:100341.

9.	Muresan S, Sitzmann M, Southan C. Mapping between databases of compounds and protein targets. Methods Mol Biol. 2012;910:145–164.

10.	Schmidt AF, Finan C, Gordillo-Marañón M, Asselbergs FW, Freitag DF, Patel RS, et al. Genetic drug target validation using Mendelian randomisation. Nat Commun. 2020;11:3255.

11.	Gordillo-Marañón M, Zwierzyna M, Charoen P, Drenos F, Chopade S, Shah T, et al. Validation of lipid-related therapeutic targets for coronary heart disease prevention using human genetics. Nat Commun. 2021;12:6120.

12.	Storm CS, Kia DA, Almramhi MM, Bandres-Ciga S, Finan C, International Parkinson’s Disease Genomics Consortium (IPDGC), et al. Finding genetically-supported drug targets for Parkinson’s disease using Mendelian randomization of the druggable genome. Nat Commun. 2021;12:7342.

13.	Dudbridge F. Power and predictive accuracy of polygenic risk scores. PLoS Genet. 2013;9:e1003348.

14.	Burgess S, Thompson SG, CRP CHD Genetics Collaboration. Avoiding bias from weak instruments in Mendelian randomization studies. Int J Epidemiol. 2011;40:755–764.

15.	Patel A, Ye T, Xue H, Lin Z, Xu S, Woolf B, et al. MendelianRandomization v0.9.0: updates to an R package for performing Mendelian randomization analyses using summarized data. Wellcome Open Res. 2023;8:449.

16.	Gudjonsson A, Gudmundsdottir V, Axelsson GT, Gudmundsson EF, Jonsson BG, Launer LJ, et al. A genome-wide association study of serum proteins reveals shared loci with common diseases. Nat Commun. 2022;13:480.

17.	Sun BB, Maranville JC, Peters JE, Stacey D, Staley JR, Blackshaw J, et al. Genomic atlas of the human plasma proteome. Nature. 2018;558:73–79.

18.	Robins C, Liu Y, Fan W, Duong DM, Meigs J, Harerimana NV, et al. Genetic control of the human brain proteome. Am J Hum Genet. 2021;108:400–410.

19.	Wallace C. Eliciting priors and relaxing the single causal variant assumption in colocalisation analyses. PLoS Genet. 2020;16:e1008720.

20.	Hukerikar N, Hingorani AD, Asselbergs FW, Finan C, Schmidt AF. Prioritising genetic findings for drug target identification and validation. Atherosclerosis. 2024;390:117462.

21.	Zuber V, Grinberg NF, Gill D, Manipur I, Slob EAW, Patel A, et al. Combining evidence from Mendelian randomization and colocalization: Review and comparison of approaches. Am J Hum Genet. 2022;109:767–782.

22.	Giambartolomei C, Vukcevic D, Schadt EE, Franke L, Hingorani AD, Wallace C, et al. Bayesian test for colocalisation between pairs of genetic association studies using summary statistics. PLoS Genet. 2014;10:e1004383.

23.	Guo H, Fortune MD, Burren OS, Schofield E, Todd JA, Wallace C. Integration of disease association and eQTL data using a Bayesian colocalisation approach highlights six candidate causal genes in immune-mediated diseases. Hum Mol Genet. 2015;24:3305–3313.

24.	Bartova L, Fugger G, Dold M, Kautzky A, Swoboda MMM, Rujescu D, et al. The choice of either quetiapine or aripiprazole as augmentation treatment in a European naturalistic sample of patients with major depressive disorder. Int J Neuropsychopharmacol. 2022;25:118–127.

25.	Standeven LR, McEvoy KO, Osborne LM. Progesterone, reproduction, and psychiatric illness. Best Pract Res Clin Obstet Gynaecol. 2020;69:108–126.

26.	Block TS, Kushner H, Kalin N, Nelson C, Belanoff J, Schatzberg A. Combined analysis of mifepristone for psychotic depression: Plasma levels associated with clinical response. Biol Psychiatry. 2018;84:46–54.

27.	Linsen F, Broeder C, Sep MSC, Verhoeven JE, Bet PM, Penninx BWJH, et al. Glucocorticoid Receptor (GR) antagonism as disease-modifying treatment for MDD with childhood trauma: protocol of the RESET-medication randomized controlled trial. BMC Psychiatry. 2023;23:331.

28.	Huang J, Fan X, Jin X, Teng L, Yan N. Dual-pocket inhibition of Nav channels by the antiepileptic drug lamotrigine. Proc Natl Acad Sci U S A. 2023;120:e2309773120.

29.	Prabhavalkar KS, Poovanpallil NB, Bhatt LK. Management of bipolar depression with lamotrigine: an antiepileptic mood stabilizer. Front Pharmacol. 2015;6:242.

30.	Gerlach PA, Milind N, Spence JP, Pritchard JK. High false sign rates in transcriptome-wide association studies. bioRxiv. 2025.

31.	Zheng P, Pan C, Zhou C, Liu B, Wang L, Duan S, et al. Contribution of Nischarin/IRAS in CNS development, injury and diseases. J Adv Res. 2023;54:43–57.

32.	Smith KL, Jessop DS, Finn DP. Modulation of stress by imidazoline binding sites: implications for psychiatric disorders. Stress. 2009;12:97–114.

33.	Yan S, Xu C, Yang M, Zhang H, Cheng Y, Xue Z, et al. The expression of agmatinase manipulates the affective state of rats subjected to chronic restraint stress. Neuropharmacology. 2023;229:109476.

34.	[Zhu M-Y, Wang W-P, Huang J, Feng Y-Z, Regunathan S, Bissette G. Repeated immobilization stress alters rat hippocampal and prefrontal cortical morphology in parallel with endogenous agmatine and arginine decarboxylase levels. Neurochem Int. 2008;53:346–354.](http://paperpile.com/b/pkJPHs/OvQGa)

35.	[Zhu M-Y, Wang W-P, Cai Z-W, Regunathan S, Ordway G. Exogenous agmatine has neuroprotective effects against restraint-induced structural changes in the rat brain. Eur J Neurosci. 2008;27:1320–1332.](http://paperpile.com/b/pkJPHs/8927O)

36.	Gawali NB, Bulani VD, Gursahani MS, Deshpande PS, Kothavade PS, Juvekar AR. Agmatine attenuates chronic unpredictable mild stress-induced anxiety, depression-like behaviours and cognitive impairment by modulating nitrergic signalling pathway. Brain Res. 2017;1663:66–77.

37.	Kõks S, Luuk H, Nelovkov A, Areda T, Vasar E. A screen for genes induced in the amygdaloid area during cat odor exposure. Genes Brain Behav. 2004;3:80–89.

38.	Taksande BG, Kotagale NR, Tripathi SJ, Ugale RR, Chopde CT. Antidepressant like effect of selective serotonin reuptake inhibitors involve modulation of imidazoline receptors by agmatine. Neuropharmacology. 2009;57:415–424.

39.	Rahangdale S, Fating R, Gajbhiye M, Kapse M, Inamdar N, Kotagale N, et al. Involvement of agmatine in antidepressant-like effect of HMG-CoA reductase inhibitors in mice. Eur J Pharmacol. 2021;892:173739.

40.	Keller B, García-Sevilla JA. Dysregulation of IRAS/nischarin and other potential I1-imidazoline receptors in major depression postmortem brain: Downregulation of basal contents by antidepressant drug treatments. J Affect Disord. 2017;208:646–652.
